# Biotherapies in Solid Tumors: Are Negative Results Still of Low Priority for Publication?

**DOI:** 10.3389/fonc.2018.00062

**Published:** 2018-03-14

**Authors:** Alessandro Ottaiano, Antonino Cassata, Monica Capozzi, Chiara De Divitiis, Alfonso De Stefano, Antonio Avallone

**Affiliations:** ^1^Department of Abdominal Oncology, Istituto Nazionale Tumori Fondazione G. Pascale (IRCCS), Naples, Italy

**Keywords:** biotherapies, publication bias, solid tumors, survey, methodology

## Abstract

In 2005, we performed the largest survey on clinical trials of biotherapies for all solid tumors and found indirect evidence of a publication bias: editors of medical journals were more prone to publish positive results independently from the quality of the studies. We collected data from 2003 to 2015 in 487 studies, and the publication bias previously described was not found in the years between 2010 and 2015: this could be related to changes and/or innovations in the guidelines and editorial policies of oncology journals occurred over the last years.

In 2005, we performed the largest survey on clinical trials of biologic and immunologic therapies (i.e., Biotherapy: inhibitors of the proliferative signals, antiangiogenic agents, differentiating agents, inhibitors of metastasis/invasiveness, drugs with multiple mechanisms of action; Immunotherapy: vaccines, cytokines, adoptive immunotherapy, antibodies stimulating the immune system, drugs with multiple immunologic mechanisms of action) for all solid tumors in order to review their content and quality (1); we found an indirect evidence of publication bias (2) as an attitude of high-impact factor medical journals to prefer the publication of positive results. We showed that publication was related to positive results independently from study quality; the observation period ranged from 1990 to 2002. This kind of publication bias (related to the reporting of positive outcome) suggested that negative results had a lower chance of being published, particularly in high-impact factor journals.

In non-oncologic research areas, there are direct and indirect pieces of evidence that this type of publication bias is increasing (3–5). This phenomenon might reduce the visibility of negative results and affect the clinical development of a drug (identification of clinical endpoints, selection of patients and therapies, management of toxicities) because this process depends on the interaction between positive and negative findings. Furthermore, oncologic research may be affected by other publication bias previously described (6–8).

Since then, we continued to collect data about biotherapies in solid tumors (general study characteristics, patient and disease factors, study methodology and quality) from selected oncology journals limiting our search to phase II/III clinical trials in colorectal, lung, breast, prostate cancer, pancreas, and melanoma from 2003 to 2015 through Medline searching. Four hundred eighty-seven clinical trials were identified. Quality of studies (quality index: QI) was determined through a modified *ad hoc* questionnaire (Table 1). Study evaluation methods were similar to the previous study (1); however, the impact factor was arbitrarily divided into three categories: <6, 6–10, >10. A QI<50 points was calculated for 36.7% of phase II and 28.2% for phase III or II/III studies. Results were defined positive when the authors showed significant improvements in activity and/or efficacy of a new treatment, negative when it was inactive or toxic or failed to show significant ameliorations vs the referral drugs.

**Table 1:** Quality assessment questionnaire for phase II and III studies.

**A. Patient selection**
Zero points if patients are not selected on a validated biological/molecular basis; 10 points if they are
**B. Trial size**
Phase II: 2 points if total number of patients is <30; 6 points if >30 or <50; 10 points if >50
Phase III: 2 points if total number of patients is <100; 6 points if >100 or <300; 10 points if >300
Analysis and measurement of biological effect
**C. Study design**
Zero points if not reported; 10 points if yes
**D. Biological endpoints**
Zero points if not reported; 10 points if evaluations of biological endpoints are planned
**E. Response**
Zero points if criteria for response assessment are not reported; 10 points if yes
**F. Toxicity**
Zero points if criteria for toxicity evaluation are not reported; 10 points if yes
**G. PERI**^a^
Zero points if preclinical evidence is not cited; 2 points if PEI<15; 8 points if PEI>15
**H. Sites of disease**
Zero points if not reported; 8 points if reported
**I. Compliance to treatment**
Zero points if not reported; 8 points if reported
**J. Time-to-event descriptions**
Eight points if overall-survival and/or disease-free survival are reported
**K. Follow-up**
Eight points if any information about follow-up is reported (duration and lost to follow-up)

*^a^PERI (Preclinical Evidence Reporting Index) is the sum of the impact factors of the journals cited by the authors when discussing the preclinical background*.

Here, we report on the association between quality, conclusions, and other explanatory variables of the studies and impact factor of the journals in recent years. In particular, the association between positive outcome and publication in high-impact factor journals was not found in the studies published between 2010 and 2015 (202 studies). In a multiple regression model performed to analyze the correlation between impact factor and some different explanatory variables of studies [quality (<50 vs ≥50), phase of the study (II vs III), academic/company supported (yes vs no), accrual time (≤3 years vs >3 years), study conclusions (positive vs negative)] the only variable found to be predictive of publication in a high-impact factor journal between 2010 and 2015 was the quality of the study (*P* = 0.012) and not the positive result (*P* = 0.178) (Table 2).

**Table 2:** Multiple regression to analyze the relationship between explanatory variables of studies and publication in high-impact factor journals between 2010 and 2015.

Variable	No. of studies	Coefficient	SE	*R*_partial_	*P*
Quality	<50:50; >50:152	0.72	0.25	0.68	0.012
Company driven	Yes: 121; No: 81	0.36	0.11	0.14	0.081
Accrual time	<3 years: 158; >3 years: 44	0.28	0.16	0.19	0.172
Study conclusion	Positive: 137; Negative: 65	0.45	0.19	0.15	0.226
Phase	II: 142; III or II/III: 60	0.33	0.21	0.09	0.198

One possible explanation of this specific outcome-related publication bias reduction may be related to changes and/or innovations in the guidelines and editorial policies (reception, evaluation, and publication of studies with negative results) of oncology journals occurred over the last years. Our report has some limitations: (i) it represents an indirect evidence about publication bias since the “true publication bias” is the difference between published and “unpublished” studies (i.e., discordance between trial in official registries and published trial) and (ii) our QI has not been independently validated by other research groups despite being reasonably a useful tool to resume the most important methodology characteristics of a clinical study.

New generation targeted therapies are changing the therapeutic management of advanced tumors and the interest on studying new therapeutic pathways as well as new drugs is increasing. We show an indirect evidence that editors and reviewers have improved in past few years their overall methodology when discussing and evaluating clinical studies on biotherapies.

## Author Contributions

AO contributed in planning, analysis, discussing, and writing of this manuscript. AC and MC contributed in discussing and writing of the manuscript. CD and AS contributed in analysis and writing of the manuscript. AA contributed in discussing of the manuscript.

## Conflict of Interest Statement

The authors declare that the research was conducted in the absence of any commercial or financial relationships that could be construed as a potential conflict of interest.
